# Supplementary material for: RAIN: machine learning-based identification for HIV-1 bNAbs
Source: Nat Commun. 2024 Jun 24;15:5339. doi: 10.1038/s41467-024-49676-1 (PMC11196741; doi:10.1038/s41467-024-49676-1)
Supplement: Supplementary file 1 — Supplementary Information [file 41467_2024_49676_MOESM1_ESM.pdf]

RAIN: Machine Learning-based identification for HIV-1 bNAbs

Mathilde Foglierini[1,2,3,*], Pauline Nortier[1,2,*], Rachel Schelling[1,2], Rahel R. Winiger[1,2], Philippe Jacquet[4], Sijy O'Dell[5], Davide Demurtas[6], Maxmillian Mpina[7], Omar Lweno[7], Yannick D. Muller[1,2], Constantinos Petrovas[8], Claudia Daubenberger[9,10], Mathieu Perreau[1], Nicole A Doria-Rose[5], Raphael Gottardo[3] and Laurent Perez[1,2,#]

[1]Department of Medicine, Service of Immunology and Allergy, Lausanne University Hospital and University of Lausanne, Lausanne, Switzerland.

[2]Centre for Human Immunology Lausanne, Switzerland.

[3]Biomedical Data Science Centre, Lausanne University Hospital and University of Lausanne, Lausanne, Switzerland.

[4]Scientific Computing and Research Support Unit, University of Lausanne, Lausanne, Switzerland.

[5]Vaccine Research Center, National Institute of Allergy and Infectious Diseases, National Institutes of Health, Bethesda, MD, USA.

[6]Interdisciplinary center of electron microscopy, CIME, Ecole Polytechnique Fédérale de Lausanne, Lausanne, Switzerland.

[7]Ifakara Health Institute, Bagamoyo, United Republic of Tanzania.

[8]Department of Laboratory Medicine and Pathology, Institute of Pathology, Lausanne University Hospital, Lausanne, Switzerland

[9]Department of Medical Parasitology and Infection Biology, Clinical Immunology Unit, Swiss Tropical and Public Health Institute, Basel, Switzerland.

[10]University of Basel, Basel, Switzerland.

*These authors contributed equally

#Corresponding author: Laurent Perez

email: laurent.perez@chuv.ch

Supplementary Information

Supplementary Figures 1-10
Supplementary Tables 1-3

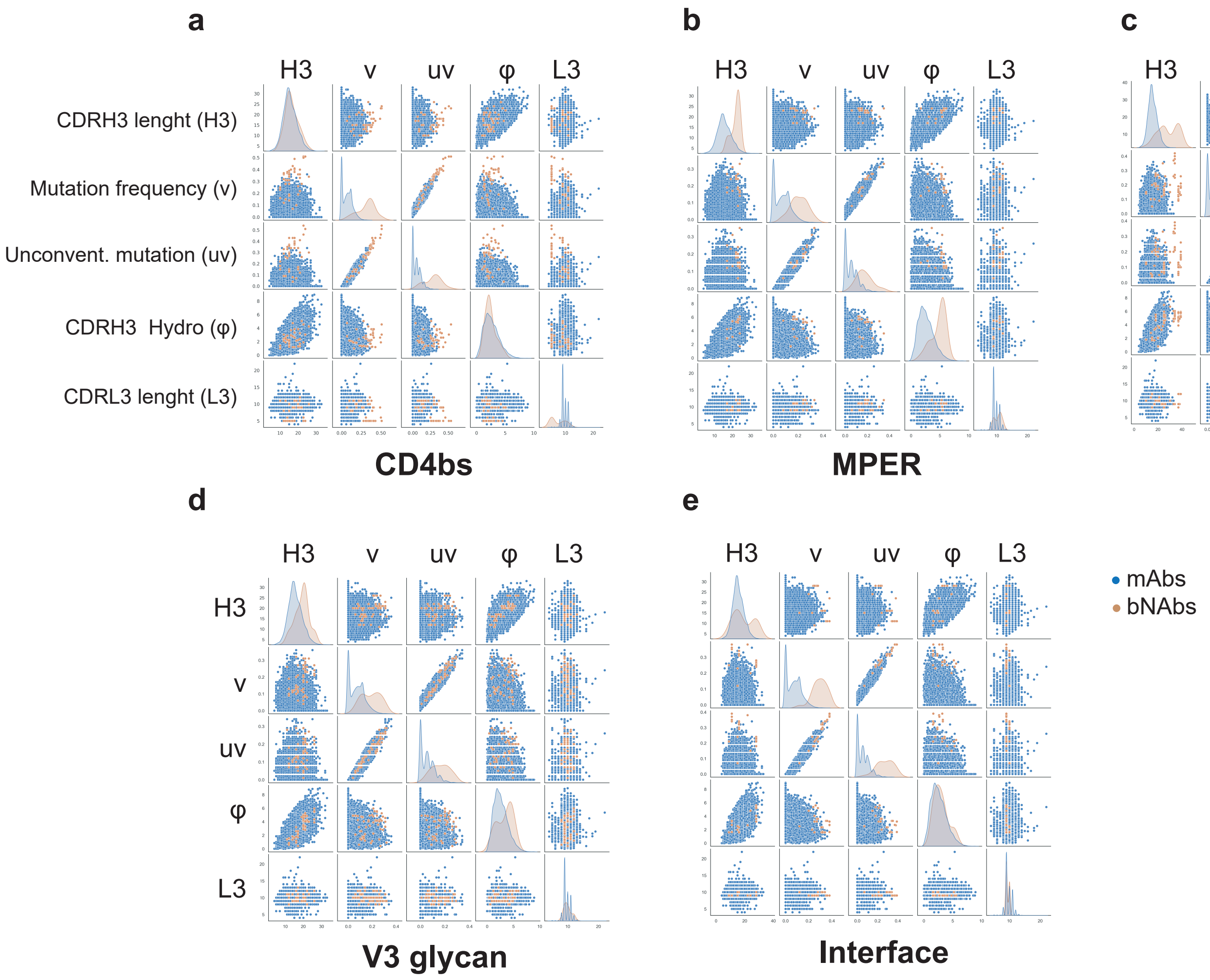

Supplementary Figure. 1. Correlation of each feature pair in the dataset against the class distribution. Pairwise relationships between the specific features comparing mAbs (blue) and bNAbs (red). Each bNAb category is represented by a single plot per antigenic site: (**a**) CD4bs, (**b**) MPER, (**c**) V1V2 apex, (**d**) V3 glycan, and (**e**) gp120/gp41 interface**.** Source data are provided as a Source Data file.

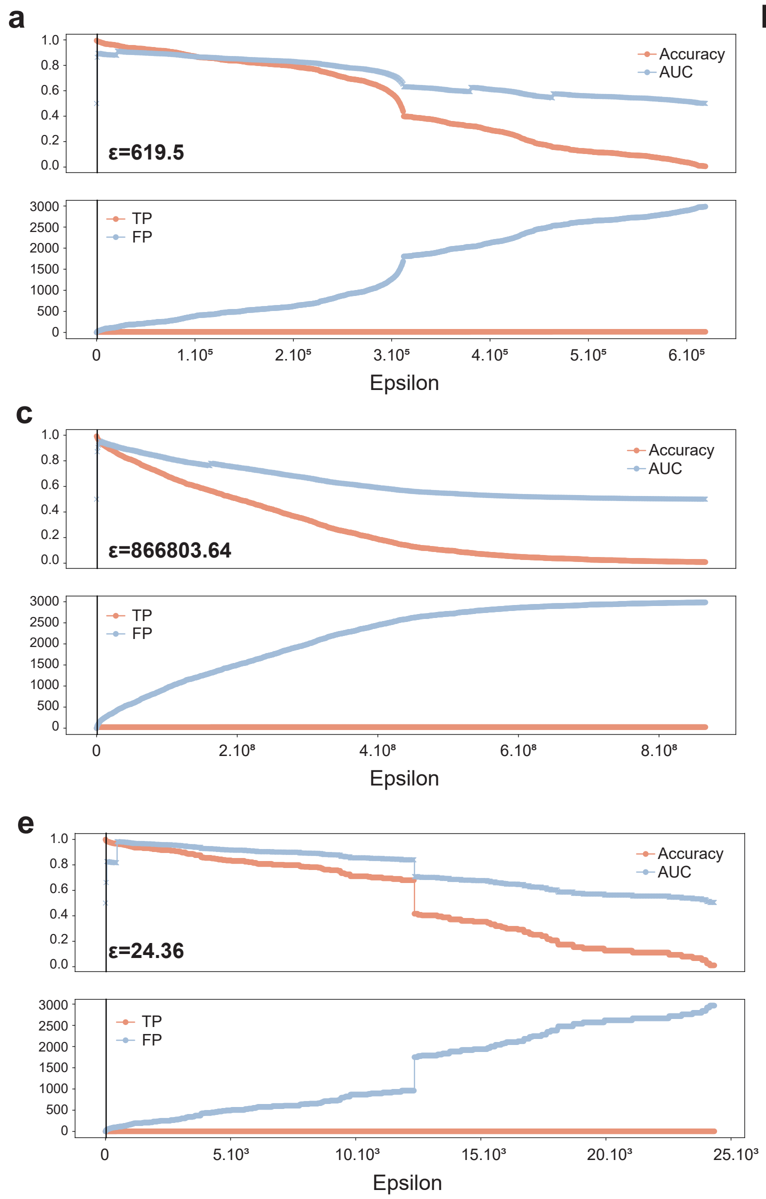

Supplementary Figure 2. Determination of the epsilon parameter for the Anomaly detection algorithm. Epsilon parameters for the antigenic site (**a**) CD4bs, (**b**) MPER, (**c**) V1V2 apex, (**d**) V3 glycan, and (**e**) gp120/gp41 interface. The accuracy is in red and the area under the curve (AUC) is in blue.

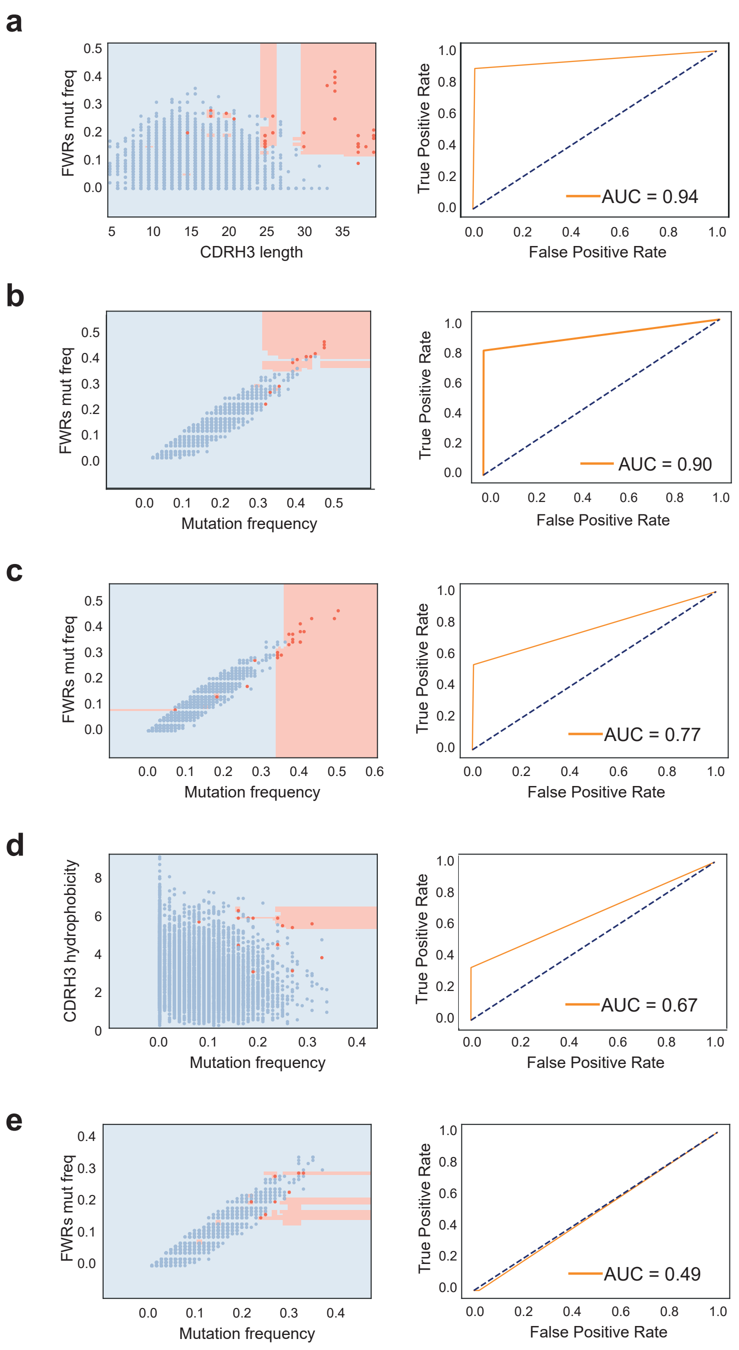

Supplementary Figure 3. Random Forest classifier with only two features. Left panels are the decision regions obtained from the training dataset and right panels are the receiver-operating characteristic (ROC) curves in orange with the corresponding area under the curve (AUC). The dashed blue line is for visual reference.
Statistics are visualized for the different antigenic sites: **(a)** V1V2 apex, **(b)** gp120/gp41 interface**, (c)** CD4bs**, (d)** MPER, and **(e) V**3 glycan.

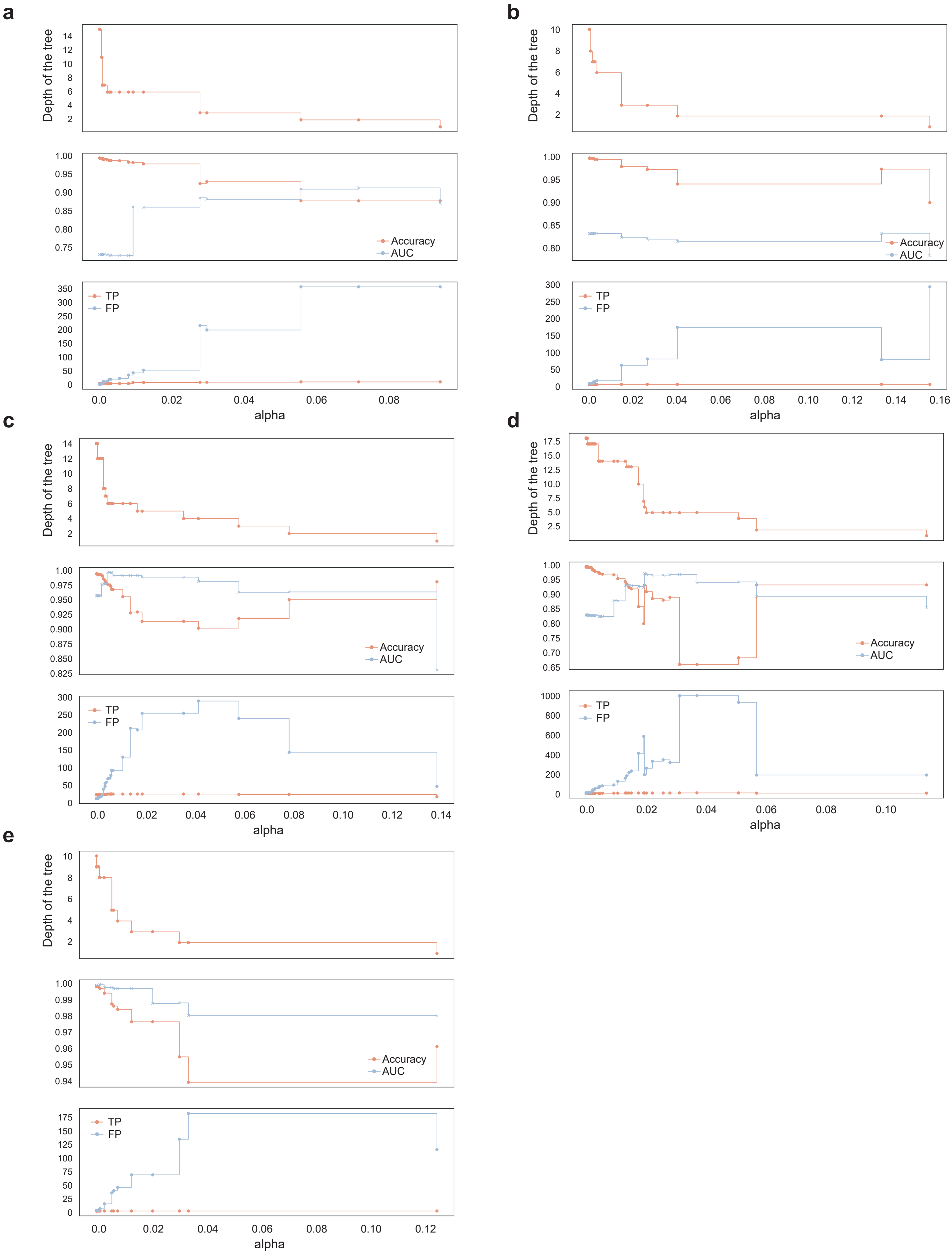

Supplementary Figure 4. Determination of the cost complexity pruning parameter alpha (ccp_alpha) for the Decision Tree classifier. Targeted antigenic sites are **(a)** CD4bs, **(b)** MPER**, (c)** V1V2 apex, **(d)** V3 glycan, and **(e)** gp120/gp41 interface.

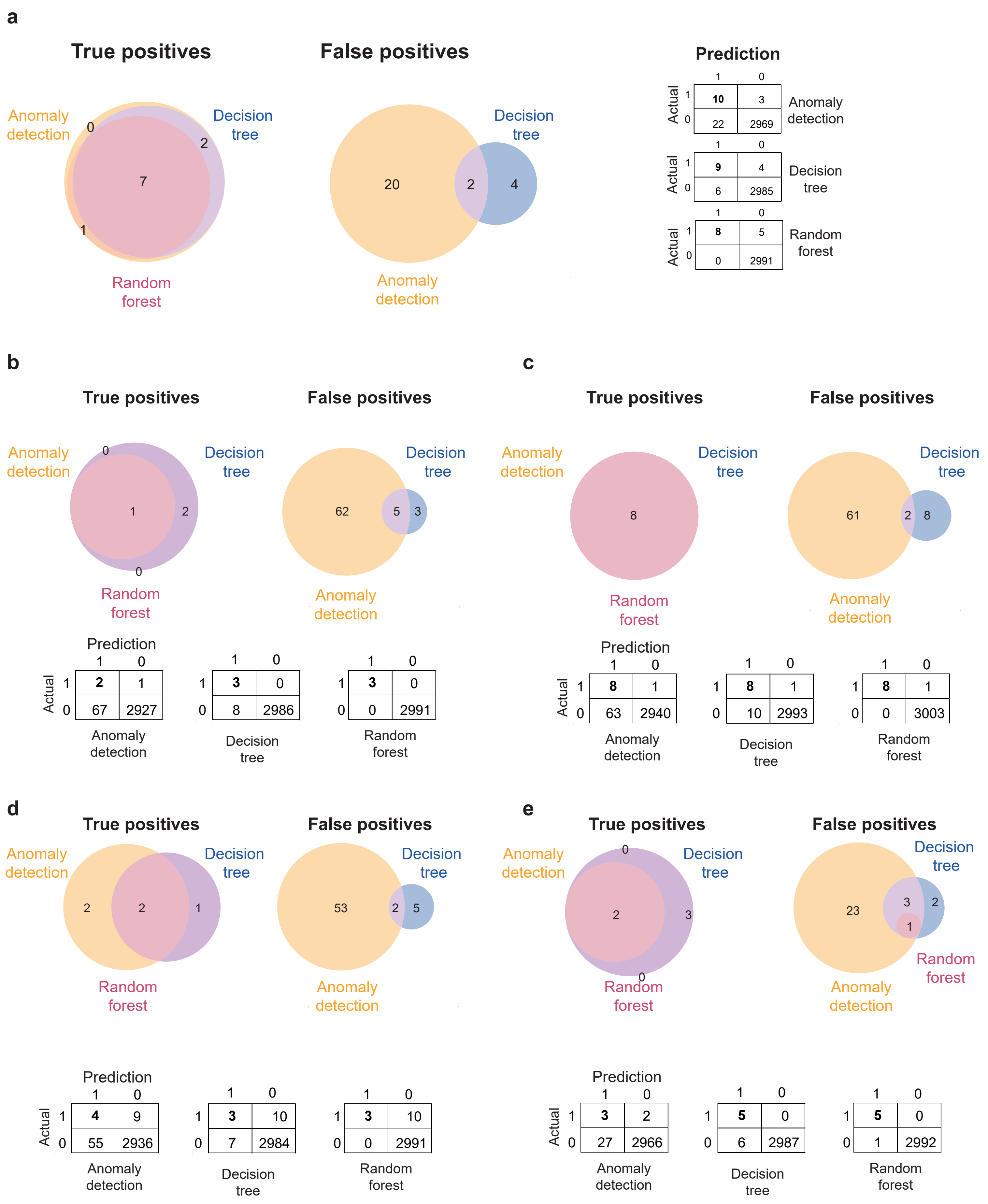

Supplementary Figure 5. Overlap of false and true positives between the three different models using the test datasets. Venn diagrams and Confusion matrices for each model are represented next to the Venn diagram. Antigenic sites are **(a)** CD4bs, **(b)** MPER, **(c)** V1V2 apex, **(d)** V3 glycan, and **(e)** gp120/gp41 interface. The yellow circle represents AD, the blue one is DT, and the red one is RF. Source data are provided as a Source Data file.

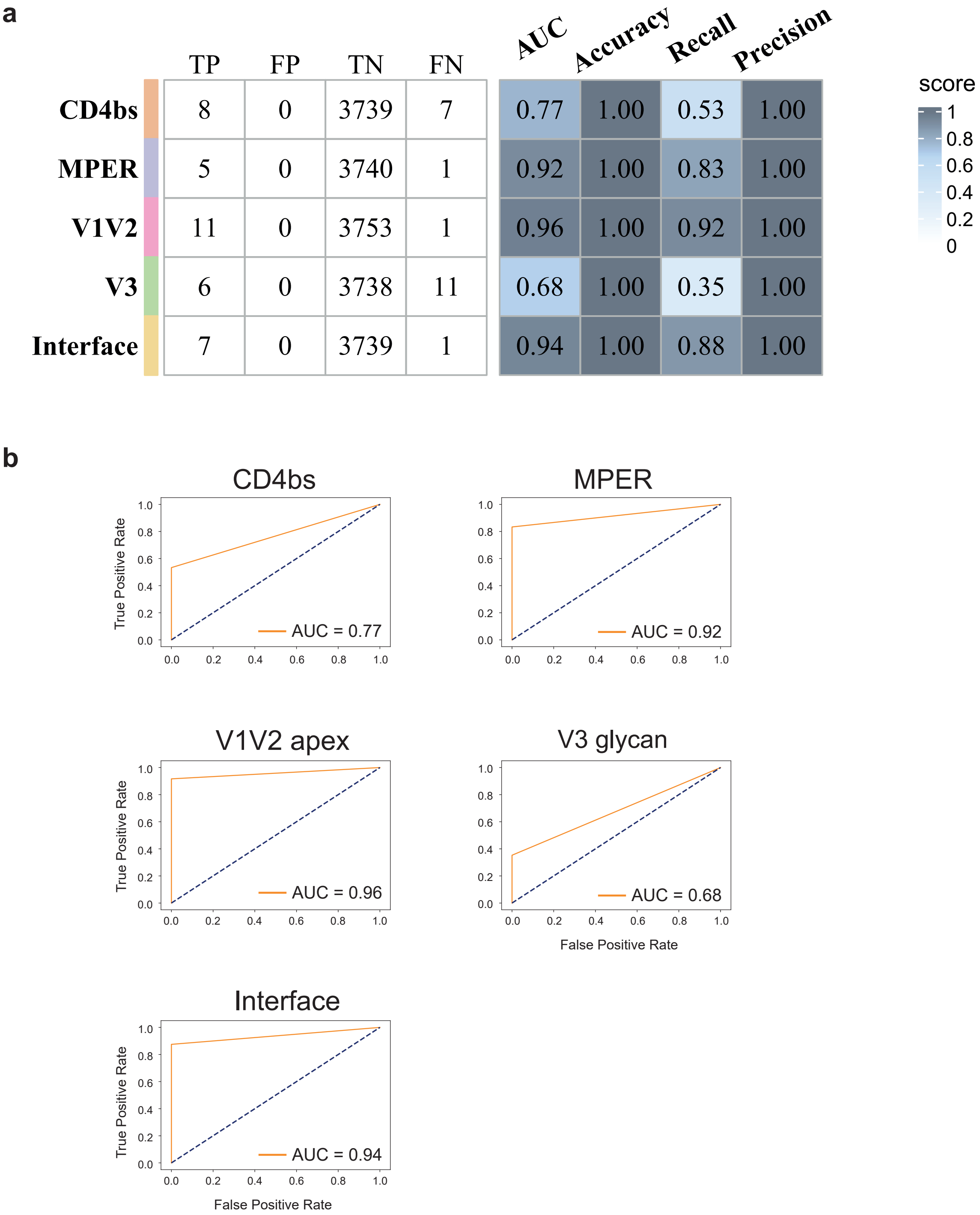

Supplementary Figure 6. Performance and results of the Super Learner Ensembles algorithm. **(a)** Performance metrics of the algorithm using the test dataset with Accuracy = (TP+TN) / (TP+FP+TN+FN), Recall = TP / (TP+FN), and Precision = TP / (TP+FP). **(b)** Receiver-operating characteristic (ROC) curves and corresponding area under the curve (AUC) statistics for each bNAb antigenic site with the test dataset.

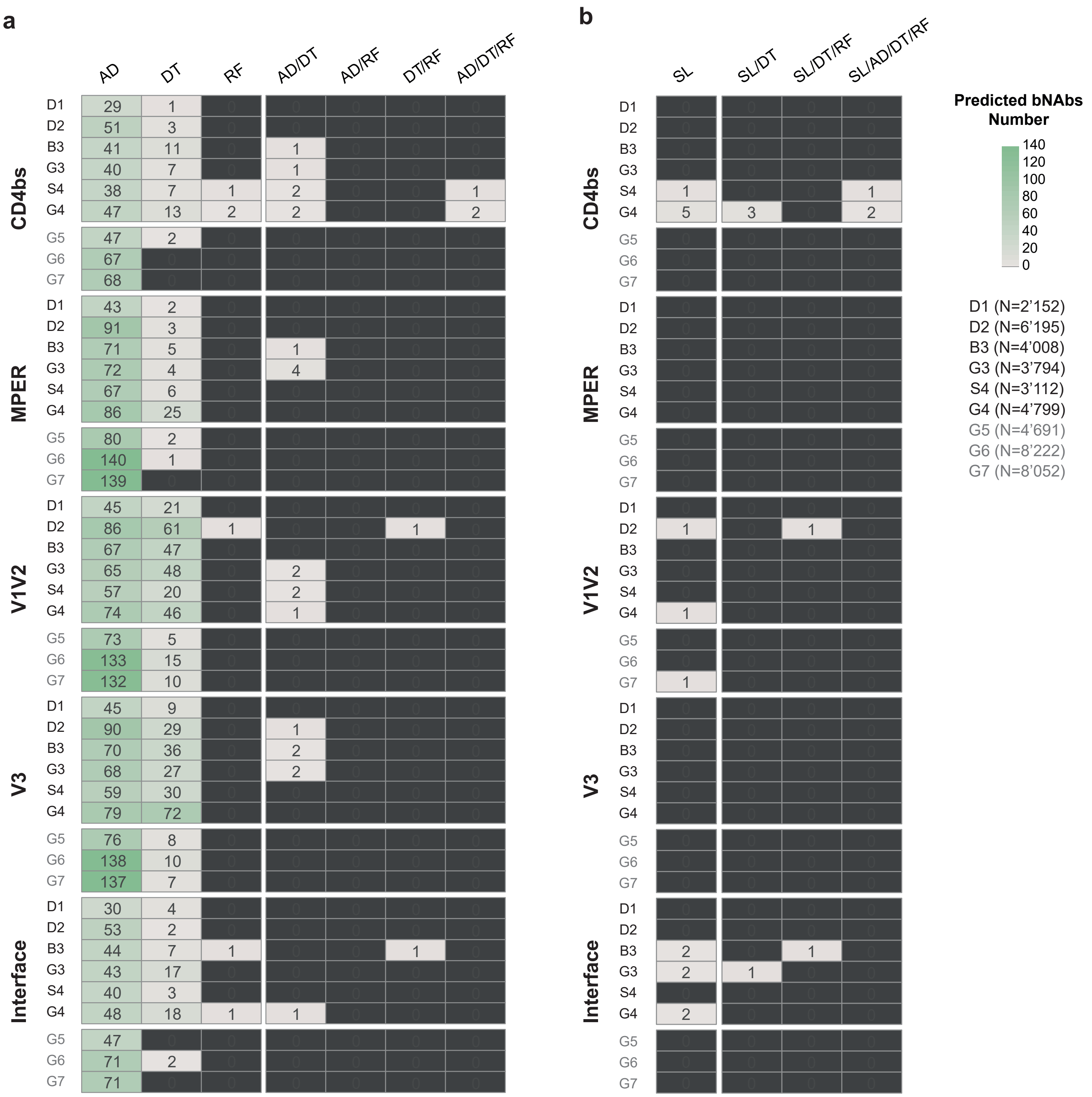

Supplementary Figure 7. Predicted bNAbs per dataset. (a) The heatmap illustrates the number of predicted bNAbs for each antigenic site and each run of donors using various algorithms, with D1: donor 1, D2: donor 2 (donor that did not have sera with broad neutralization activity) and B3, G3, S4, G4: donor 3 (donor serum with broad neutralization activity). The G5, G6, and G7 datasets correspond to a Influenza-specific repertoire.
The columns on the right side of the heatmap show bNAbs identified by different algorithms. The abbreviations used in each cell represent the combination of algorithms that share the specific bNAbs (AD/DT: bNAbs predicted only by both AD and DT, AD/RF: bNAbs predicted only by both AD and RF, DT/RF: bNAbs predicted only by both DT and RF and AD/DT/RF: bNAbs predicted by all AD, DT and RF). **(b)** The heatmap illustrates the number of predicted bNAbs for each antigenic site and each run of donors using the Super Learner (SL) algorithm.
The columns on the right side of the heatmap show bNAbs shared by different algorithms. The abbreviations used in each cell represent the combination of algorithms that share the specific bNAbs (SL/DT: bNAbs predicted only by both SL and DT, SL/DT/RF: bNAbs predicted only by SL, DT and RF and SL/AD/DT/RF: bNAbs predicted by all SL, AD, DT and RF).

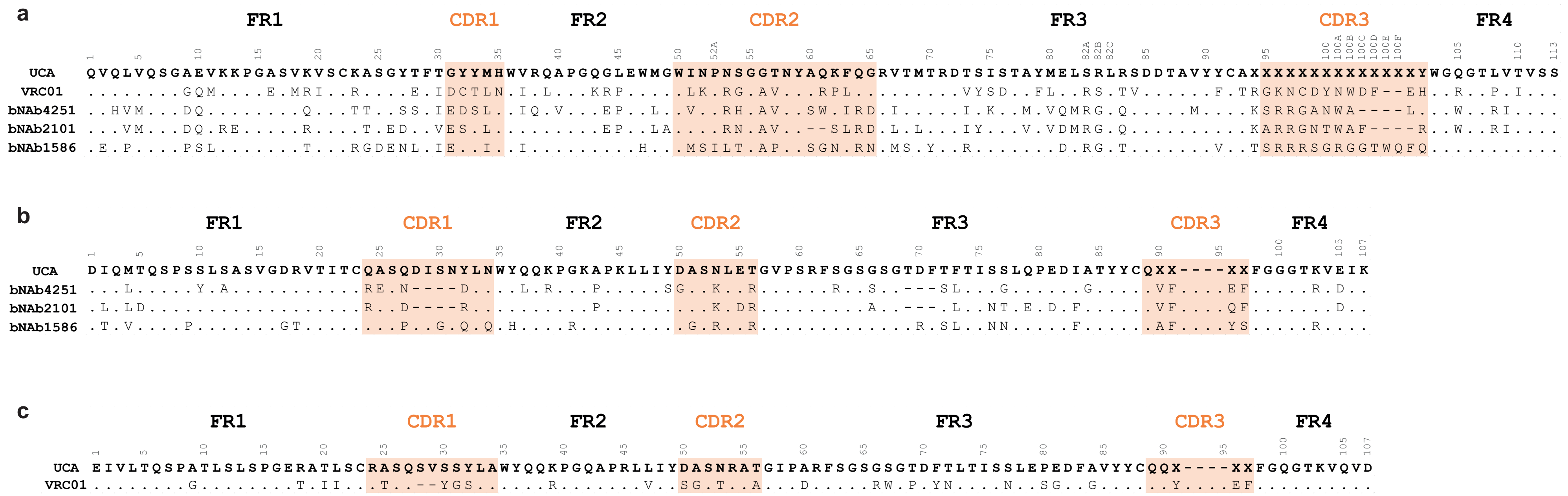

Supplementary Figure 8. bNAbs alignment of VH and VK amino acid sequences. Upper panel: (**a**) Alignment of the VH sequence of the three predicted bNAbs with their UCA (unmutated common ancestor) and VRC01 bNAb. UCA is constituted of VH1-2*02 and JH4*02, the D gene is masked and represented by X. Residue positions are according to Kabat numbering. Dots indicate identical residues. (**b**) Middle panel: alignment of the VK sequences of the three predicted bNAbs with their UCA (unmutated common ancestor). UCA is constituted of VK1-33*01 and JK4*01, the LCDR3 is masked and represented by X. (**c**) Lower panel: alignment of the VK sequence of the VRC01 with its UCA VK3-11*01 and JK2*01.

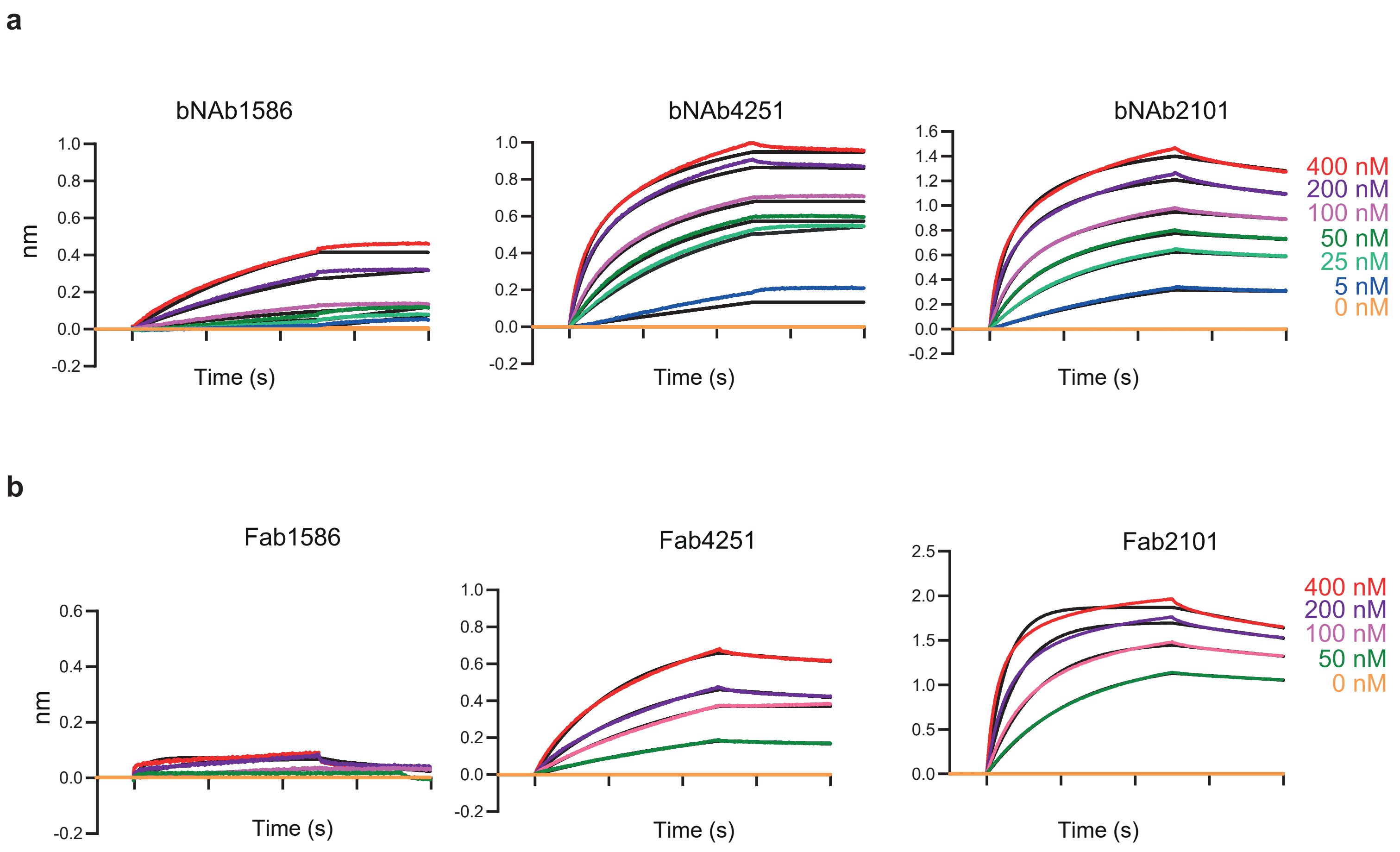

Supplementary Figure 9. Antibodies and Fabs interaction with BG505-DS-SOSIP.
(a) Bivalent analyte fitting for antibody SOSIP interactions with concentrations ranging from 5 to 400 nM**.**
(b) Fitting of Fab interaction with BG505-DS-SOSIP using 1:1 model. For Fabs, concentrations ranging from 50 to 400 nM were used.Source data are provided as a Source Data file.

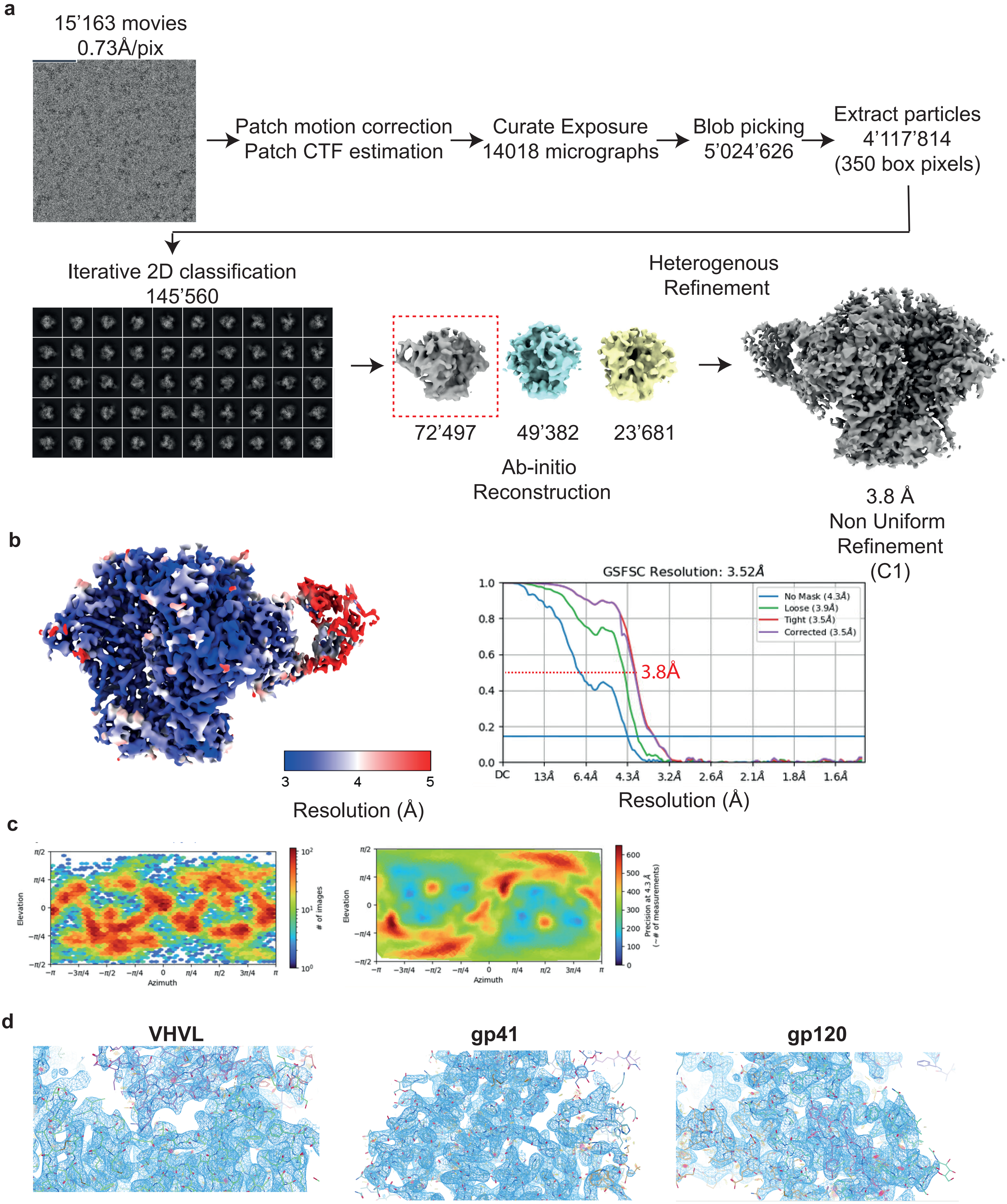

Supplementary Figure 10. Cryo-EM data processing and validation of Fab-BG505-DS-SOSIP complexes.
(a) Representative cryo-EM micrograph, 2D class average images and data processing flow chart.
(b) Local resolution of the final map and Gold standard Fourier shell correlation (FSC) at 0.143 resolution of 3.8 Å.
(c) Angular distribution of the Fab-BG505-DS-SOSIP particles in the final round of 3D refinement.
(d) Diferent density maps shown at threshold of 6 σ for VHVL, gp41 and gp120, respectively.

Supplementary Table 1. Source of the ten datasets used as mAbs in the machine learning models and the three datasets used as Influenza repertoires.

	Paired BCRs (IGK/L+IGH)	Reference
**H1**	4 581	ArrayExpress: E-MTAB-11174 (Memory B cells of donor 1)
**H2**	4 740	ArrayExpress: E-MTAB-11174 (Memory B cells of donor 2)
**H3**	965	SRA: SRR17717616
**H4**	903	SRA: SRR17717597
**H5**	895	SRA: SRR17717612
**H6**	413	SRA: SRR17717605
**H7**	341	SRA: SRR17717601
**H8**	786	SRA: SRR17717593
**H9**	429	10X dataset: 10k Human PBMCs, 5' v2.0, Chromium Controller, Single Cell Immune Profiling Dataset by Cell Ranger 6.1.0 (2021, August 9)
**H10**	909	10X dataset: Human PBMC from a Healthy Donor, 10k cells (v2), Single Cell Immune Profiling Dataset by Cell Ranger 5.0.0 (2020, November 19)
**G5**	4 691	BioSample SAMN07733010, Day 9 after influenza vaccination. SRA : SRR10596386, SRR10596375, SRR10596364, SRR10596353
**G6**	8 222	BioSample SAMN07733010, Day 7 after influenza vaccination. SRA : SRR10596411, SRR10596412, SRR10596410, SRR10596409
**G7**	8 052	BioSample SAMN07733010, Day 7 after influenza vaccination. SRA : SRR10596407, SRR10596406, SRR10596405, SRR10596404

Supplementary Table 2. Performance metrics of the three algorithms using the validation dataset.

Algo	Ag site	bNAbs Numbers	TP	FP	TN	FN	AUC	Accuracy	Recall	Precision
**AD**	CD4bs	15	12	22	2966	3	0.90	0.99	0.80	0.35
**DT**	CD4bs	15	7	4	2984	8	0.73	1.00	0.47	0.64
**RF**	CD4bs	15	7	0	2988	8	0.90	1.00	0.47	1.00
**AD**	MPER	3	1	38	2956	2	0.66	0.99	0.33	0.03
**DT**	MPER	3	2	4	2990	1	0.83	1.00	0.67	0.33
**RF**	MPER	3	2	0	2994	1	0.83	1.00	0.67	1.00
**AD**	V1V2 apex	25	19	55	2932	6	0.87	0.98	0.76	0.26
**DT**	V1V2 apex	25	23	12	2975	2	0.96	1.00	0.92	0.66
**RF**	V1V2 apex	25	19	0	2987	6	1.00	1.00	0.76	1.00
**AD**	V3 glycan	9	5	57	2938	4	0.77	0.98	0.56	0.08
**DT**	V3 glycan	9	6	8	2987	3	0.83	1.00	0.67	0.43
**RF**	V3 glycan	9	2	0	2995	7	1.00	1.00	0.22	1.00
**AD**	Interface	3	1	31	2964	2	0.66	0.99	0.33	0.03
**DT**	Interface	3	3	4	2991	0	1.00	1.00	1.00	0.43
**RF**	Interface	3	2	0	2995	1	1.00	1.00	0.67	1.00

Supplementary Table 3. Cryo-EM data collection, refinement, and validation statistics.

Data Accession PDB ID: 8S2E EMDB: EMD-19665	
Magnification	165'000
Voltage (kV)	300
Electron exposure (e–/Å^2)	39.89
Defocus range (μm)	-0.9 to -2.4
Pixel size (Å)	0.73277
Symmetry imposed	C1
Micrographs collected (no.)	15,163
Final particle images (no.)	72497
Map resolution (Å)	3.8
FSC threshold	0.143
Map resolution range (Å)	
**Refinement**	
Initial model used (PDB code)	4TVP
Model resolution (Å)	3.8
FSC threshold	0.143
Map sharpening *B* factor (Å^2)	
Model composition	
Non-hydrogen atoms	16305
Protein residues	1958
Ligands	BMA:6
	NAG:73
	MAN:6
B factors (Å^2)	
Protein	100.6
R.m.s. deviations	
Bond lengths (Å)	0
Bond angles (°)	0.75
Validation	
MolProbity score	2.10
Clashscore	9.0
Poor rotamers (%)	2.3
Ramachandran plot	
Favored (%)	95.5
Allowed (%)	4.5
Disallowed (%)	0.0
