# Supplementary material for: RAIN: machine learning-based identification for HIV-1 bNAbs
Source: Nat Commun. 2024 Jun 24;15:5339. doi: 10.1038/s41467-024-49676-1 (PMC11196741; doi:10.1038/s41467-024-49676-1)
Supplement: Supplementary file 2 — Reporting Summary [file 41467_2024_49676_MOESM2_ESM.pdf]

nature portfolio

Corresponding author(s): Laurent Perez

Last updated by author(s): Jun 6, 2024

Reporting Summary

Nature Portfolio wishes to improve the reproducibility of the work that we publish. This form provides structure for consistency and transparency in reporting. For further information on Nature Portfolio policies, see our Editorial Policies and the Editorial Policy Checklist.

Statistics

For all statistical analyses, confirm that the following items are present in the figure legend, table legend, main text, or Methods section.

n/a	Confirmed	
☐	☒	The exact sample size (*n*) for each experimental group/condition, given as a discrete number and unit of measurement
☐	☒	A statement on whether measurements were taken from distinct samples or whether the same sample was measured repeatedly
☐	☒	The statistical test(s) used AND whether they are one- or two-sided<br>*Only common tests should be described solely by name; describe more complex techniques in the Methods section.*
☐	☒	A description of all covariates tested
☐	☒	A description of any assumptions or corrections, such as tests of normality and adjustment for multiple comparisons
☐	☒	A full description of the statistical parameters including central tendency (e.g. means) or other basic estimates (e.g. regression coefficient) AND variation (e.g. standard deviation) or associated estimates of uncertainty (e.g. confidence intervals)
☐	☒	For null hypothesis testing, the test statistic (e.g. *F*, *t*, *r*) with confidence intervals, effect sizes, degrees of freedom and *P* value noted<br>*Give P values as exact values whenever suitable.*
☒	☐	For Bayesian analysis, information on the choice of priors and Markov chain Monte Carlo settings
☐	☒	For hierarchical and complex designs, identification of the appropriate level for tests and full reporting of outcomes
☒	☐	Estimates of effect sizes (e.g. Cohen's *d*, Pearson's *r*), indicating how they were calculated

Our web collection on statistics for biologists contains articles on many of the points above.

Software and code

Policy information about availability of computer code

Data collection	FACSAria Fusion sorter (BD), ChromiumTM Controller (10x genomics),BD Rhapsody Single-Cell Analysis System (BD Biosciences).<br>Biolayer interferometry (BLI) data were acquired using Octet R2 (Sartorius).<br>The cryo-EM data was collected by using EPU software on Titan Krios, equipment with Falcon IV detector in electron counting mode.
Data analysis	BLI data analysis was performed using the Octet software and GraphPad Prism (Version 9.0)<br>CryoEM analysis was done with cryoSPARC (Version 4.4), Coot (V0.9.8.8) and Phenix (Version 1.21).<br>Structure display ChimeraX Version 1.5 and Pymol (Version 4.6).<br>FACS:BD FACSDiva (version .9.0) software, FlowJo (v.10.7.1).<br>Cellranger v6.0.0 and v7.0.1 for the preprocessing of the raw 10X V(D)J data.<br>BD Rhapsody Targeted mRNA Analysis Pipeline (version 1.11) for the BD Rhaspody V(D)J and targeted gene expression data.<br>Celescope v1.14.1 for the Singleron V(D)J data followed by cellranger v7.0.1.<br>IgBlast v1.17.1 for the annotation of the VDJ sequences followed by MakeDB.py from change-O package (Immcantation pipeline).<br>ANARCI for the identification of the junction region for the BCR protein sequences coming from CATNAP database.<br>R Statistical Software (v4.2.1) for the data processing, graphing and statistical analysis (with ggstatsplot package).<br>Machine Learning algorithms with Python v3.8.16 and scikit-learn package v1.0.2.<br>All codes available at https://github.com/MathildeFogPerez/manuscript-bnab-foglierini

For manuscripts utilizing custom algorithms or software that are central to the research but not yet described in published literature, software must be made available to editors and reviewers. We strongly encourage code deposition in a community repository (e.g. GitHub). See the Nature Portfolio guidelines for submitting code & software for further information.

Data

Policy information about availability of data

All manuscripts must include a data availability statement. This statement should provide the following information, where applicable:

- Accession codes, unique identifiers, or web links for publicly available datasets
- A description of any restrictions on data availability
- For clinical datasets or third party data, please ensure that the statement adheres to our policy

The authors declare that all data suporting the findings are available within the manuscript and VDJ sequencing are available at GEO database: GSE229123. Cryo-EM map was deposited with EMD-19665 and PDB number 8S2E.

Research involving human participants, their data, or biological material

Policy information about studies with human participants or human data. See also policy information about sex, gender (identity/presentation), and sexual orientation and race, ethnicity and racism.

Reporting on sex and gender	Blood samples were collected from adults (N=25, 16 females and 9 males).
Reporting on race, ethnicity, or other socially relevant groupings	Race and ethnicity information were not collected.
Population characteristics	Participants included in the study were adults who provided written informed consent and were defined as having HIV infection based on a rapid serological test (Alere Determine HIV-1/2 test).
Recruitment	Adult participants with HIV infection providing a written informed consent were recruted at Ifakara Health Institute Institutional for blood donation.
Ethics oversight	Approved by the ethics committee EKBB; Basel, Switzerland; reference number 342/10<br>Ifakara Health Institute Institutional 619 Review Board (Reference number IHI/IRB/No.24-2010)

Note that full information on the approval of the study protocol must also be provided in the manuscript.

Field-specific reporting

Please select the one below that is the best fit for your research. If you are not sure, read the appropriate sections before making your selection.

[x] Life sciences [ ] Behavioural & social sciences [ ] Ecological, evolutionary & environmental sciences

For a reference copy of the document with all sections, see nature.com/documents/nr-reporting-summary-flat.pdf

Life sciences study design

All studies must disclose on these points even when the disclosure is negative.

Sample size	PBMCs from all 25 donors were collectected and no statistical methods were used to predetermine sample size.
Data exclusions	No data are excluded
Replication	Experiments were at least repeated twice.
Randomization	No randomization was performed.
Blinding	Neutralisation assay were performed blind.

Reporting for specific materials, systems and methods

We require information from authors about some types of materials, experimental systems and methods used in many studies. Here, indicate whether each material, system or method listed is relevant to your study. If you are not sure if a list item applies to your research, read the appropriate section before selecting a response.

Materials & experimental systems

n/a	Involved in the study
☐	☒ Antibodies
☐	☒ Eukaryotic cell lines
☒	☐ Palaeontology and archaeology
☒	☐ Animals and other organisms
☒	☐ Clinical data
☒	☐ Dual use research of concern
☒	☐ Plants

Methods

n/a	Involved in the study
☒	☐ ChIP-seq
☒	☐ Flow cytometry
☒	☐ MRI-based neuroimaging

Antibodies

Antibodies used	CD20-PE-Cy7 (clone L27, catalog no. 335793, BD 637 Biosciences)<br>F(ab')2-Goat anti-Human IgG Fc secondary antibody, APC (RRID: AB_2337695, Jackson ImmunoResearch).
Validation	Validation is available on the manufacturer´s website.

Eukaryotic cell lines

Policy information about cell lines and Sex and Gender in Research

Cell line source(s)	Expi293 cells (ThermoFisher Cat No. A14527), TZM-bl (NIBSC, ARP5011)
Authentication	Done by the manufacturer´s.
Mycoplasma contamination	Cells were regularly tested negative for mycoplama.
Commonly misidentified lines<br>(See ICLAC register)	No commonly misidentified cell line have been used in this study.

Plants

Seed stocks	NA
Novel plant genotypes	NA
Authentication	NA
