# Supplementary material for: RAIN: machine learning-based identification for HIV-1 bNAbs
Source: Nat Commun. 2024 Jun 24;15:5339. doi: 10.1038/s41467-024-49676-1 (PMC11196741; doi:10.1038/s41467-024-49676-1)
Supplement: Supplementary file 4 — Description of Additional Supplementary Files [file 41467_2024_49676_MOESM4_ESM.pdf]

Supplementary Data 1. Curated list of Immunoglobulin Paired Receptors from the CATNAP Database.

This dataset includes HIV-1 paired immunoglobulins used for machine learning (ML) applications, listed according to their CATNAP ID, donor, and associated antigenic regions. Additional information provided includes V and J gene usage, insertion and mutation frequencies, amino acid sequences of the VH/VL and CDR3 regions. When available, the PDB code, clonal lineage, PubMed ID, and the number of viruses tested are also indicated.
